# Characterization of Zymosan-Modulated Neutrophils With Neuroregenerative Properties

**DOI:** 10.3389/fimmu.2022.912193

**Published:** 2022-05-30

**Authors:** Andrew D. Jerome, Jeffrey R. Atkinson, Arnetta L. McVey Moffatt, Jesse A. Sepeda, Benjamin M. Segal, Andrew R. Sas

**Affiliations:** ^1^Department of Neurology, Ohio State Medical Center, Columbus, OH, United States; ^2^Neuroscience Research Institute, The Ohio State University, Columbus, OH, United States

**Keywords:** neutrophils, regeneration, heterogeneity, transcriptomics, proteomics

## Abstract

Recent studies using advanced techniques such as single cell RNA sequencing (scRNAseq), high parameter flow cytometry, and proteomics reveal that neutrophils are more heterogeneous than previously appreciated. Unique subsets have been identified in the context of bacterial and parasitic infections, cancer, and tissue injury and repair. The characteristics of infiltrating neutrophils differ depending on the nature of the inflammation-inciting stimulus, the stage of the inflammatory response, as well as the tissue microenvironment in which they accumulate. We previously described a new subpopulation of immature Ly6G^low^ neutrophils that accumulate in the peritoneal cavity 3 days following intraperitoneal (i.p.) administration of the fungal cell wall extract, zymosan. These neutrophils express markers of alternative activation and possess neuroprotective/regenerative properties. In addition to inducing neurite outgrowth of explanted neurons, they enhance neuronal survival and axon regeneration *in vivo* following traumatic injury to the optic nerve or spinal cord. In contrast, the majority of neutrophils that accumulate in the peritoneal fluid 4 hours following i.p. zymosan injection (4h NΦ) have features of conventional, mature Ly6G^hi^ neutrophils and lack neuroprotective or neuroregenerative properties. In the current study, we expand upon on our previously published observations by performing a granular, in-depth analysis of these i.p. zymosan-modulated neutrophil populations using scRNAseq and high parameter flow cytometry. We also analyze cell lysates of each neutrophil population by liquid chromatography/mass spectrometry. Circulating blood neutrophils, harvested from naive mice, are analyzed in parallel as a control. When samples were pooled from all three groups, scRNAseq revealed 11 distinct neutrophil clusters. Pathway analyses demonstrated that 3d NΦ upregulate genes involved in tissue development and wound healing, while 4h NΦ upregulate genes involved in cytokine production and perpetuation of the immune response. Proteomics analysis revealed that 3d NΦ and 4h NΦ also express distinct protein signatures. Adding to our earlier findings, 3d NΦ expressed a number of neuroprotective/neuroregenerative candidate proteins that may contribute to their biological functions. Collectively, the data generated by the current study add to the growing literature on neutrophil heterogeneity and functional sub-specialization and might provide new insights in elucidating the mechanisms of action of pro-regenerative, neuroprotective neutrophil subsets.

## 1 Introduction

It has long been understood that lymphocyte and monocytes/macrophages can be polarized, both *in vitro* and *in vivo*, into functional subsets with distinctive transcriptome signatures and cell surface phenotypes (1–4). In contrast, mature peripheral neutrophils have traditionally been viewed as a homogenous population of terminally differentiated, granulocytic cells with highly conserved physiological functions (5). They typically form the first line of defense against pathogenic invaders and acute tissue injury (5, 6). In addition to clearing microbes, neutrophils have been implicated in anti-tumor responses, autoimmunity, and wound healing (7–12). In keeping with this wide range of functional activities, recent analyses of neutrophils at the single cell level have shown them to be more plastic and diverse than previously appreciated. Neutrophil subsets differ in cell surface and cytoplasmic protein expression, and transcriptomic profiles, that correlate with their biological properties, and reflect their environmental exposures, tissue localization and maturation/activation state. Characteristic subsets have been identified across tissues during homeostasis, as well as in the setting of bacterial and parasitic infections, cancer, and tissue injury (12–16). Newly identified neutrophil populations include alternatively activated cells that suppress anti-tumor immune responses, modulate inflammation during chronic infections, and promote tissue repair after cardiac injury (9, 16, 17).

Traditionally, central nervous system (CNS) infiltrating neutrophils have been portrayed as playing a detrimental role in the aftermath of traumatic brain and spinal cord injury or stroke. Neutrophils have been held responsible for initiating a destructive inflammatory cascade within the CNS, mediated by the release of reactive oxygen species (ROS), matrix metalloproteases (MMPs), and chemokines, following stimulation with danger-associated molecular pattern signals (DAMPS) (18). We recently described a new subset of immature (Ly6G^low^, C101^neg^) murine neutrophils with ring shaped nuclei that possess neuroprotective and neuroregenerative properties (16). In contrast to conventional pro-inflammatory neutrophils, they exhibit features of alternative activation, including expression of CD206, and IL-4 receptor α (IL4rα) chain transcripts. Large numbers of these alternatively activated neutrophils accumulate in the peritoneal cavity 3 days following intraperitoneal (i.p.) administration of the fungal cell wall extract, zymosan (16). In addition to inducing neurite outgrowth of explanted dorsal root ganglion and retinal neurons, they enhance neuronal survival and axon regeneration *in vivo* following traumatic injury to the optic nerve or spinal cord, in part *via* secretion of a panel of growth factors including insulin-like growth factor-1 (IGF-1) and nerve growth factor (NGF) (16). In contrast, the majority of neutrophils that accumulate in the peritoneal fluid 4 hours following i.p. zymosan injection (4h NΦ) display features of conventional, mature Ly6G^hi^ neutrophils and lack neuroprotective or neuroregenerative properties. In the current study, we compare the cell surface phenotypes, transcriptomic, and proteomic profiles of zymosan-modulated 4h NΦ and 3-day neutrophils (3d NΦ) with each other, as well as with naïve circulating syngeneic neutrophils.

## 2 Materials and Methods

Mice. C57BL/6 WT male mice aged 8-10 weeks were purchased from the Jackson Laboratory. Mice were group-housed with a 12-h light/dark cycle and *ad libitum* access to food and water. All animal handling and surgical procedures were performed in compliance with national guidelines and approved by the Ohio State University Committees on Use and Care of Animals.

### 2.1 Intraperitoneal Lavage and Neutrophil Isolation From Blood and Peritoneal Cavity 

*I.P. Neutrophil isolation:* Mice were injected i.p. with 500 μL of zymosan solution (2μg/μL in PBS) and euthanized either 4 hours or 3 days later *via* CO_2_ fixation. 10 ml of sterile ice-cold PBS was injected into the peritoneal cavity, then aspirated after 5 minutes. All i.p. zymosan stimulated neutrophils were harvested between 12 pm and 1 pm.

*Blood neutrophil isolation:* Mice were euthanized by isoflurane overdose. Blood was collected from the left ventricle in an EDTA tube. PBMC were isolated over a lympholyte gradient (Cedarlane labs), and incidental red blood cells were lysed using an Ammonium-Chloride-Potassium (ACK) Lysing Buffer (Quality Biological). To ensure these naïve neutrophils be consistent with the i.p. zymosan stimulated neutrophils, these neutrophils were isolated at 12 pm as well.

*Neutrophil isolation:* Neutrophils were isolated from the i.p. lavaged cells using MACS Ly6G positive selection magnetic beads (Miltenyi Biotec) following the manufacturer’s instructions. Purity (95-99%) was verified *via* flow cytometric analysis.

### 2.2 Flow Cytometry

Flow cytometric analysis was performed as previously described (16). Cells were labeled with fixable viability dye (eFluor506 or eFluor780; eBioscience), blocked with anti-CD16/32 (clone 2.4G2), and stained with fluorochrome-conjugated antibodies specific for CD11b (clone M1/70), Ly6G (1A8), CD45 (30-F11), IL4ra (mIL4R-M1), and CD14 (rmC5-3), all purchased from BD Pharmingen. Fluorochrome-conjugated antibodies specific for CD101 (polyclonal), and CD62L (MEL-14) were purchased from eBiosciences. CXCR2 (SA044G4) was purchased from Biolegend. For intracellular staining, cells were fixed and permeabilized with BD cytofix and cytoperm solutions, then stained with fluorescent antibodies specific for CD206 (MR6F3; eBiosciences). Flow cytometry was performed with a FACS Symphony A3 cell analyzer (BD Biosciences). Cells were gated on forward and side scatter after doublet exclusion and analyzed on FlowJo v10 software (**Supplemental Figure 1A**).

### 2.3 Single Cell RNA Sequencing Analysis

Neutrophils were purified as described above. Cell purity and viability was confirmed by flow cytometry (**Supplemental Figure 1B**). Single cell RNA sequencing was performed as previously described (16). Briefly, the single-cell suspension was loaded onto a well on a 10x Chromium Single Cell instrument (10x Genomics). Barcoding and cDNA synthesis were performed according to the manufacturer’s instructions. The 10x GemCode Technology partitions thousands of cells into nanoliter-scale Gel Bead-In-EMulsions (GEMs), where all the cDNA generated from an individual cell share a common 10x Barcode. To identify the PCR duplicates, a unique molecular identifier (UMI) was also added. The GEMs were incubated with enzymes to produce full-length cDNA, which was then amplified by PCR to generate enough quantity for library construction. Qualitative analysis was performed using the Agilent Bioanalyzer High Sensitivity assay. The cDNA libraries were constructed using the 10x Chromium Single cell 3′ Library Kit according to the manufacturer’s protocol. For quality control after library construction, 1 μl of sample was diluted 1:10 and ran on the Agilent Bioanalyzer High Sensitivity chip for qualitative analysis. For quantification, an Illumina Library Quantification Kit was used. Libraries were sequenced on an Illumina HiSeq or NextSeq 2 × 150 paired-end kits using the following read lengths: 26-bp Read1 for cell barcode and UMI, 8-bp I7 index for sample index and 98-bp Read2 for transcript. Cell Ranger 1.3 (https://10xgenomics.com/) was used to process Chromium single-cell 3′ RNA-seq output (19).

The scRNA seq data was processed and visualized using the Seurat package (v.4.1.0) (20) in R (4.1.0). The data was initially filtered for quality control through the removal of individual cells that detected less than 200 genes or more than 7000. Cells with less than 20 percent mitochondrial counts were retained. Samples were normalized using sctransform. The genes used to identify the monocyte populations in the naïve samples were identified using the top 50 markers for monocytes in blood according to the Mouse Cell Atlas (21). The markers for the naïve samples were calculated and the clusters with at least 10 of the top 50 markers were removed.

Integration anchors were determined using the integration features that had selected the top 5000 features with the “SCT” normalization method. The data was integrated with all genes and the “SCT” normalization method. PCA and UMAP were run on the integrated object and then the nearest neighbors (dims= 1:30) and clusters were identified with a low resolution (0.23).

Markers for each cluster were identified using a Wilcoxon rank-sum test and corrected for multiple testing with the Bonferroni method with the Seurat FindAllMarkers function set to return the positive results with a minimum log2 Fold Change of 0.25. The top five and top one hundred genes in each cluster were selected by ordering the identified genes in each cluster by log Fold Change. Pathway enrichment analysis was performed using Gene Set Enrichment Analysis from the fgsea package (v. 4.1) with the GO Biological Process Ontology downloaded from MSigDB (22). Heatmaps were generated using ComplexHeatmap (v. 4.1) (23) with z-score transformed normalized gene expression values.

*Monocle pseudotime*: A Monocle3 cell dataset object was created from the integrated Seurat object. PCA and UMAP were run on the Monocle3 object then cells were clustered using the cluster_cells function (24). The principal graph was learned using the learn_graph function with a minimal branch length of 15 and a geodesic distance ratio of 0.5. The pseudotimes were calculated using the order_cells function after the root nodes were selected in the center of the cells that clustered in the naïve cells. Visualization was performed using the plot_cells function.

### 2.4 Mass Spectrometry Proteomic Analysis

*Protein Extraction:* Neutrophils were purified as described above. Cell lysis was performed on at least 1.5x10^6^ cells per mouse (n=3/condition) with 350 μL of urea lysis buffer (8 M urea, 50 mM Tris.HCl pH8, 150 mM NaCl, 1X Roche complete protease inhibitor) using a QSonica sonic probe with the following settings: Amplitude 50%, Pulse 10 x 1s. 1 on 1 off. The lysate was incubated at room temperature for 1 hour with mixing at 1000 rpm in an Eppendorf ThermoMixer and then the clarified by centrifugation at 10,000g for 10 minutes at 25°C. The cleared lysate was transferred to 1.5 mL LoBind Eppendorf tubes. Protein quantitation was performed using a Qubit protein assay (Invitrogen).

SDS-PAGE and Trypsin Digestion 10μg of samples was processed by SDS-PAGE using a 10% Bis Tris NuPage mini-gel (Invitrogen) in the MES buffer system. The migration windows (1 cm gel lane) were excised and processed by in-gel digestion with trypsin using a ProGest robot (DigiLab) with the following protocol: 1) Washed with 25 mM ammonium bicarbonate followed by acetonitrile. 2) Reduced with 10 mM dithiothreitol at 60°C followed by alkylation with 50 mM iodoacetamide at RT. 3) Digested with trypsin (Promega) at 37°C for 4 hours. 4) Quenched with formic acid and the supernatant was analyzed directly without further processing.

*Mass Spectrometry:* Half of each digest was analyzed by nano LC-MS/MS with a Waters NanoAcquity HPLC system interfaced to a ThermoFisher Fusion Lumos mass spectrometer. Peptides were loaded on a trapping column and eluted over a 75 μm analytical column at 350 nL/min; both columns were packed with Luna C18 resin (Phenomenex). The mass spectrometer was operated in data-dependent mode, with the Orbitrap operating at 60,000 FWHM and 15,000 FWHM for MS and MS/MS respectively. The instrument was run with a 3s cycle for MS and MS/MS. Advanced Precursor Determination was employed (25). 4 hours of instrument time was used per sample.

*Data Processing:* Data were processed with MaxQuant version 1.6.0.13 (Max Planck Institute for Biochemistry) which incorporates the Andromeda search engine (26).

## 3 Results

### 3.1 Zymosan-Modulated 4h NΦ and 3d NΦ Have Different Cell Surface Phenotypes

**Figure 1:**
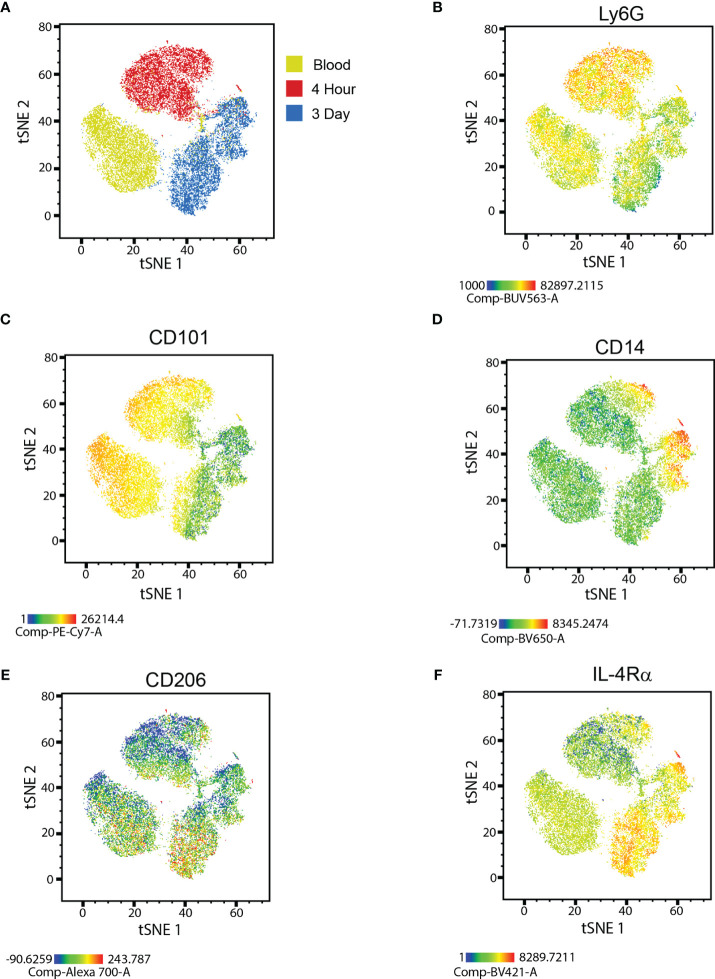
Flow cytometric characterization of neutrophils. **(A)** t-SNE plot of naïve blood, 4 hour, and 3 day i.p. zymosan stimulated neutrophils showing unique clusters for each neutrophil type. Heat map overlay of protein expression focusing on **(B)** Ly6G, **(C)** CD101, **(D)** CD14, **(E)** CD206, and **(F)** IL-4Rα in each of the clusters.

We subjected Ly6G^+^ neutrophils, isolated from the peritoneal cavity of C57BL/6 mice either 4 hours or 3 days following intraperitoneal (i.p.) zymosan injection, or from the circulation of naïve syngeneic mice to multi-parameter flow cytometry. t-Distributed Stochastic Neighbor Embedding (t-SNE) analysis, based on relative expression of cell surface markers, showed that the neutrophils fell into 3 main clusters that correspond to their source of origin (**Figure 1A**). The majority of circulating blood neutrophils and 4h NΦ expressed high levels of Ly6G and CD101, consistent with a mature stage of development. In contrast, the majority of 3d NΦ expressed lower levels of Ly6G and CD101, suggestive of immaturity (**Figures 1B, C**). The majority of 3d NΦ also expressed IL-4Rα chain and a subpopulation expressed CD206 (mannose receptor), suggestive of alternative activation; while the majority of blood and 4h NΦ were CD206 and IL4Rα negative or low (**Figures 1E, F**). Subpopulations of both 4h and 3d NΦ expressed high levels of CD14, a marker more typically associated with monocytes (**Figure 1D**). We previously reported that neuroregenerative neutrophils are CD14^+^ (16). Interestingly, a CD14^hi^ population of immunosuppressive neutrophils was recently described in tumor-bearing mice (8).

### 3.2 Zymosan-Induced i.p. Neutrophils Are Transcriptionally Diverse

**Figure 2:**
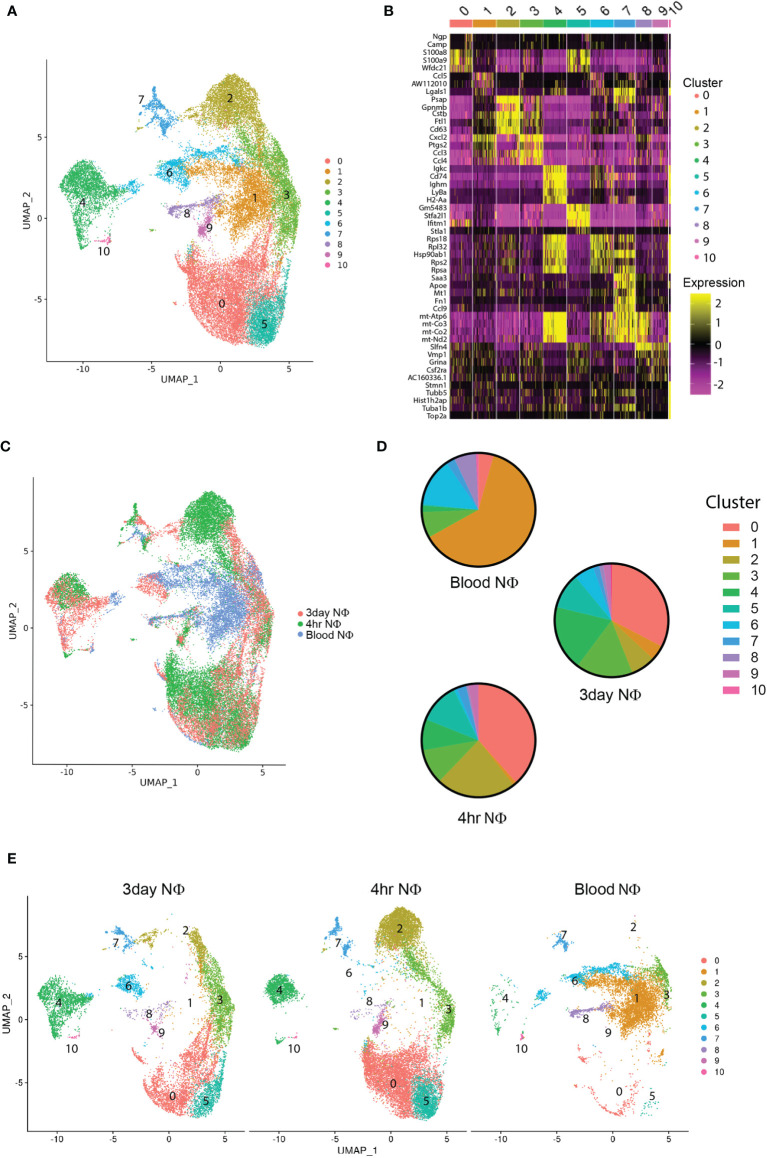
scRNA seq characterization of neutrophils. **(A)** scRNA seq of purified blood, 4 hour, and 3 day neutrophils unbiased cluster formation based on differentially expressed genes in each cluster. **(B)** Heat map of the top 5 cluster defining genes for each cluster. **(C)** Overlay of neutrophil type on the defined UMAP clusters from **(A)** showing the location of blood neutrophils, 4 hour, and 3 day i.p zymosan stimulated neutrophils. **(D)** Pie charts illustrating the percentage of total neutrophils in each cluster for blood, 4 hour, and 3 day neutrophils. **(E)** UMAP clustering analysis demonstrating the cluster distribution of all cells from each neutrophil group (blood, 4 hour, and 3 day) within the total cellular analysis of all cell groups combined.

Next, we characterized the purified Ly6G^+^ 4h NΦ, 3d NΦ, and blood neutrophils by single cell RNA sequencing (scRNAseq). These studies included two biological replicates (of cells pooled from 3 mice per replicate) in each group. Combined analysis of all 34,056 cells pooled together, identified 11 distinct clusters (**Figures 2A, B**). The majority of blood neutrophils fell into cluster 1 (62.5%) or cluster 6 (13.8%), 4h NΦ were predominantly found in cluster 0 (38.4%), cluster 2 (22.7%), and cluster 5 (11.7%), and 3d NΦ had high percentages of cells in cluster 0 (32.6%), cluster 3 (16.3%), and cluster 4 (18.5%) (**Figures 2C–E**).

**Figure 3:**
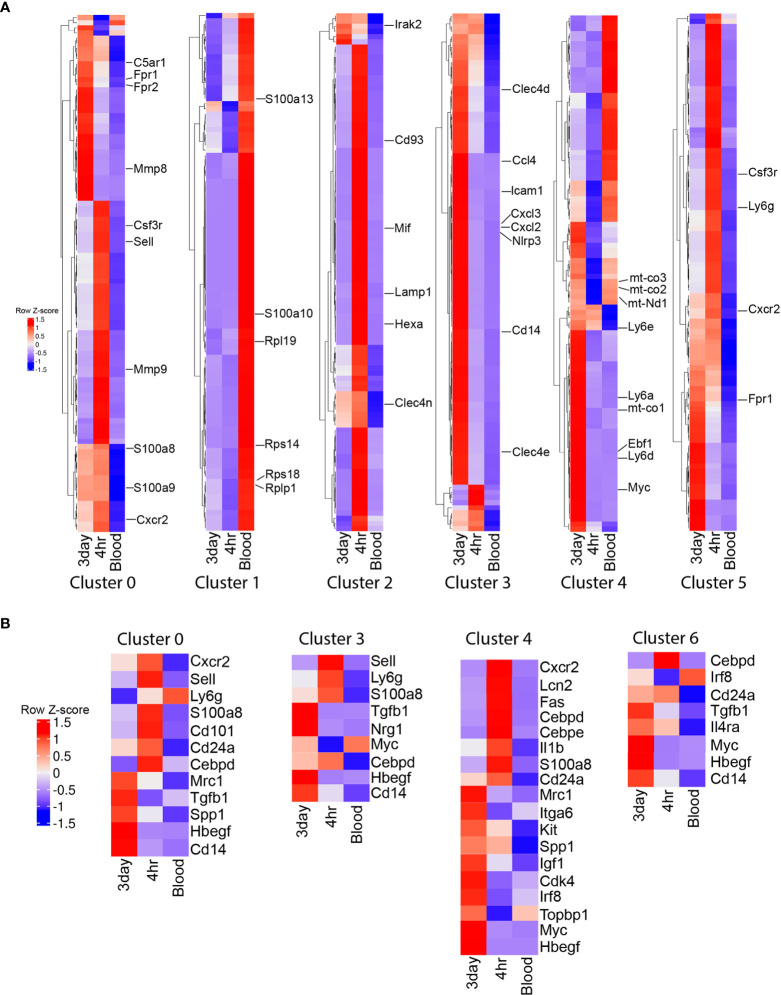
Single cell RNA sequencing cluster evaluation. **(A)** Heat map of the top 100 differentially expressed genes that define the clusters with the highest percentage of cells (0, 1, 2, 3, 4, and 5) with select genes displayed. **(B)** Heat map showing relative gene expression of genes associated with maturation and activation state across neutrophil populations within the clusters with the highest percentage of 3 day neutrophils.

To better understand the functional role of these transcriptional differences between clusters and cell types, we examined the differential expression gene ontology (GO) biological pathways and the top 100 cluster defining genes in clusters that had the highest percentages of cells. The majority of blood neutrophils fell into cluster 1 (65%), with biological processes associated with metabolism (Supplemental figure 2) and cluster defining genes associated with homeostatic markers (*S100a10, S100a13, Hmgb1)* and ribosomal function and protein synthesis (*Rps14, Rps18, Rplp1, Rpl19*) (**Figure 3A**). Cluster 0 comprises a similar percentage of cells in the 4h and 3d NΦ cohorts and was defined by biological pathways associated with inflammation and immune cell activation (**Supplemental Figure 2**). The cluster defining genes included classical activation markers of neutrophils *S100a8, S100a9, Sell, C5ar1, Csf3r, Mmp8, Mmp9*, *Fpr1, Fpr2*, and *Cxcr2* (**Figure 3A**). Cluster 2, which is most frequent in the 4h NΦ cohort (22.7%) compared to 7% of 3d NΦ, expresses genes associated with metabolism and lysosomal pathways and cluster defining genes that include relatively high levels *of Hexa, Irak2, Clec4n, Cd93, Mif*, and *Lamp1* transcripts (**Figure 3A**). Cluster 3 had higher percentages of 3d NΦ (16.3%) compared to the other neutrophil subsets with biological pathways focused on inflammation and cytokine production (**Figure 3A**, **Supplemental Figure 2**). Cluster 3 defining genes included markers of inflammation including *Icam1, Cxcl2*, *Nlrp3*, and *Il1b*, and was notable for C*d14*, a characteristic of the pro-regenerative neutrophils (16). Cluster 4 was also differentially expanded in the 3d NΦ (18.5%) compared to 4h NΦ (8%) and blood NΦ (1.8%) defined by pathways associated with metabolism (**Supplemental Figure 2**). Some of the differential genes within this cluster included markers associated with an immature cell (*Ly6a*, *Ly6d*, *Ly6e*, *Myc*, and *Ebf1)*, as well as with oxidative phosphorylation (*Mt-co1, Mt-co2, and Mt-co3*) indicating immature metabolically activated myeloid cells (27, 28) (**Figure 3A**). Cluster 5 was similar to cluster 0 in its expression of classical neutrophil maturation markers with high expression of *Ly6g, Cxcr2, Csfr3*, and *Fpr1*.

In addition to examining cluster defining genes and their relative expression across the neutrophil groups, we also examined relative expression of known genes associated with maturation and activation state within the clusters with the highest percentage of 3d NΦ (**Figure 3B**). Genes associated with neutrophil classical activation and a mature state were upregulated in 4h NΦ compared to 3d NΦ including *Ly6g, Cxcr2, Sell, CD101, Cebpd, Lcn2*, and *S100a8* in clusters 0, 3, 4, and 6 (**Figure 3B**) (13). 3d NΦ had higher expression of genes associated with an immature neutrophil state including *Myc, Topbp1, Kit*, and *Irf8* (13). Transcripts associated with alternative activation were increased in 3d NΦ compared to 4h NΦ in the clusters, including *Cd14, Tgfb1, Il4ra, Mrc1*, and *Itga6* (9, 16, 29). Additionally, across these clusters, 3d NΦ express high levels of factors associated with neuroprotection, including *Igf1, Nrg1, Hbegf*, and *Spp1*(**Figure 3B**) (16, 30–32). These changes in transcript levels associated with maturation and activation state in each cluster show that on a population level, 4h NΦ generally have a more mature, classically activated state even when they were found in clusters with enriched numbers of 3d NΦ.

**Figure 4:**
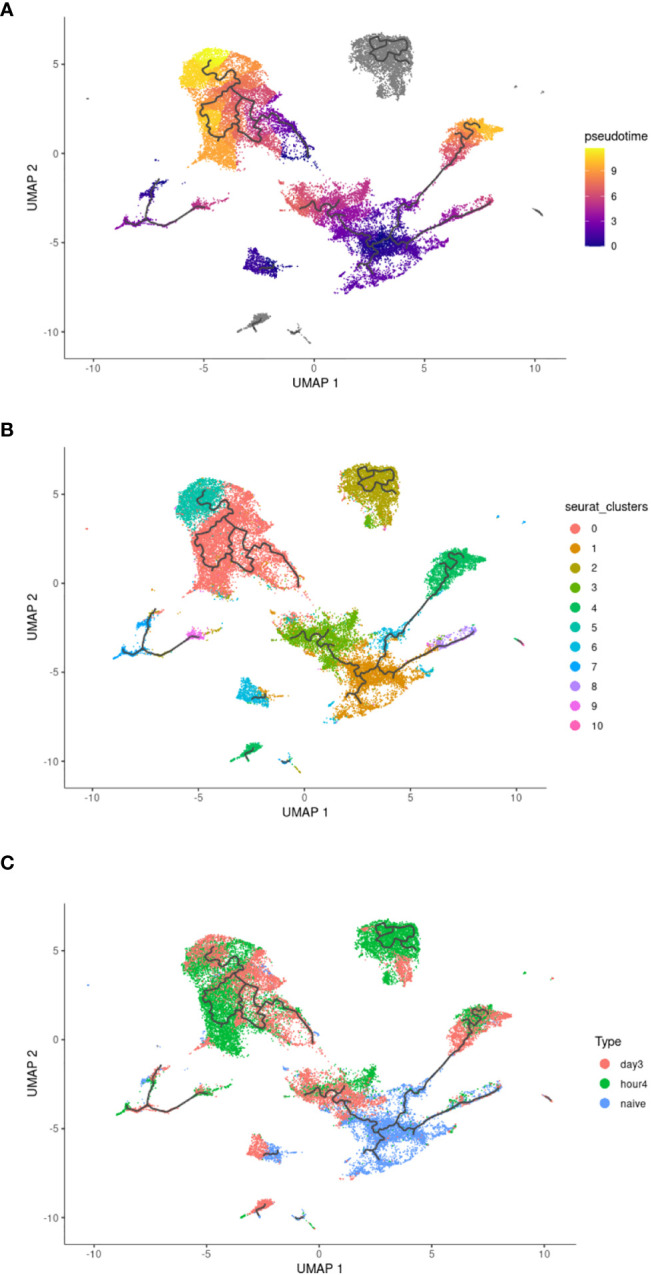
Pseudotime analysis of neutrophil clusters. **(A)** Pseudotime analysis. **(B)** Pseudotime UMAP with overlay of original clusters as defined in Figure 2A. **(C)** Distribution of each neutrophil population within the pseudotime UMAP.

To explore the transcriptional relationship of circulating blood NΦ to 4h NΦ post injection, and then 3d NΦ post injection, a pseudotime trajectory analysis of the combined samples was performed (**Figures 4A–C**). Pseudotime analysis shows the relative transition and similarities through the different clusters of neutrophils over time (**Figure 4A**). Pseudotime shows that cluster 0 and 2, which are associated with immune activation pathways are more closely related to each other and separate from cluster 1 (primarily naïve blood NΦ). Pseudotime trajectory suggests that the gene expression profile of 3d NΦ in clusters 3 and 4, which make up a significant percentage of the 3d NΦ population, separates from proinflammatory populations cluster 0 and 2 which are predominantly 4h NΦ and is more closely related to population 1 than the 4h NΦ (**Figures 4B, C**).

### 3.3 3d NΦ Upregulate Genes Associated With Tissue Development and Wound Healing, While 4h NΦ Upregulate Genes Involved in Cytokine Production and Perpetuation of the Immune Response

**Figure 5:**
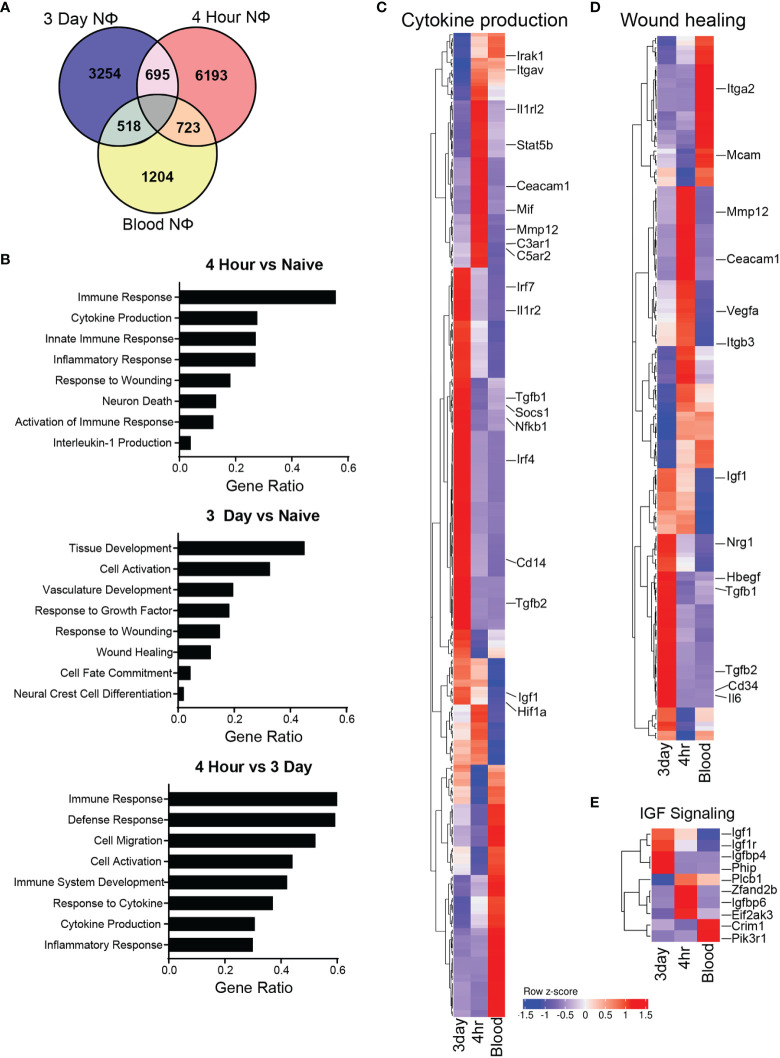
Neutrophil gene expression is unique and diverse. **(A)** Venn diagram of unique and overlapping genes between the 3 different neutrophil types. **(B)** Selected GO biological process pathway comparison based on differential expression of genes uniquely upregulated in each cell type. **(C)** Heat map of gene expression with select genes displayed from the cytokine production GO biological process pathway. **(D)** Heat map of gene expression with select genes displayed from the wound healing GO biological process pathway. **(E)** Heat map of gene expression from the insulin like growth factor GO signaling pathway between neutrophil subsets.

Next, we interrogated gene expression patterns of 3d, 4h, and circulating NΦ (agnostic to their clustering) in order to identify transcripts that were differentially expressed between those groups. There were 1204 uniquely expressed genes in the blood neutrophils, 6193 in the 4h NΦ and 3254 in the 3d NΦ compared to 723 genes shared between blood neutrophils and 4hr NΦ, 518 shared between blood and 3d NΦ, and 695 shared between 4h NΦ and 3d NΦ (**Figure 5A**). Differential expression gene ontology (GO) pathway analysis shows that 4h NΦ express higher levels of genes associated with immune response and cytokine production compared with either naïve blood or 3d NΦ (**Figure 5B**). In contrast, 3d NΦ express relatively high levels of genes associated with wound healing and regulation of the immune response (**Figure 5B**). We compared 4h, 3d, and naïve blood NΦ for the expression of individual genes associated with cytokine production or wound healing. Within the cytokine production pathway, there was upregulation of *Il1rl2, Stat5b*, *C3ar1*, and *C5ar2* in the 4hr NΦ, all of which are associated with proinflammatory signaling. In contrast, the genes identified in the cytokine production pathway that displayed increased expression in the 3d NΦ were *Tgfb1*, *Socs1*, and *Irf* genes 4, and 7, which are associated with immunoregulation (**Figure 5C**) (33, 34). In the wound healing pathway, there was an increase in expression of growth factors *Igf1, Nrg1*, *Hbegf*, and growth factor transcription factor *Egr*, which are all associated with neuroprotective effects, in the 3d NΦ compared to the 4hr or blood NΦ (**Figure 5D**) (16, 30, 35). 3d NΦ also expressed high levels of a number of genes associated with IGF1 signaling (**Figure 5E**).

### 3.4 Proteomic Analysis Characterizes the Pro-Regenerative Neutrophil Proteome

**Figure 6:**
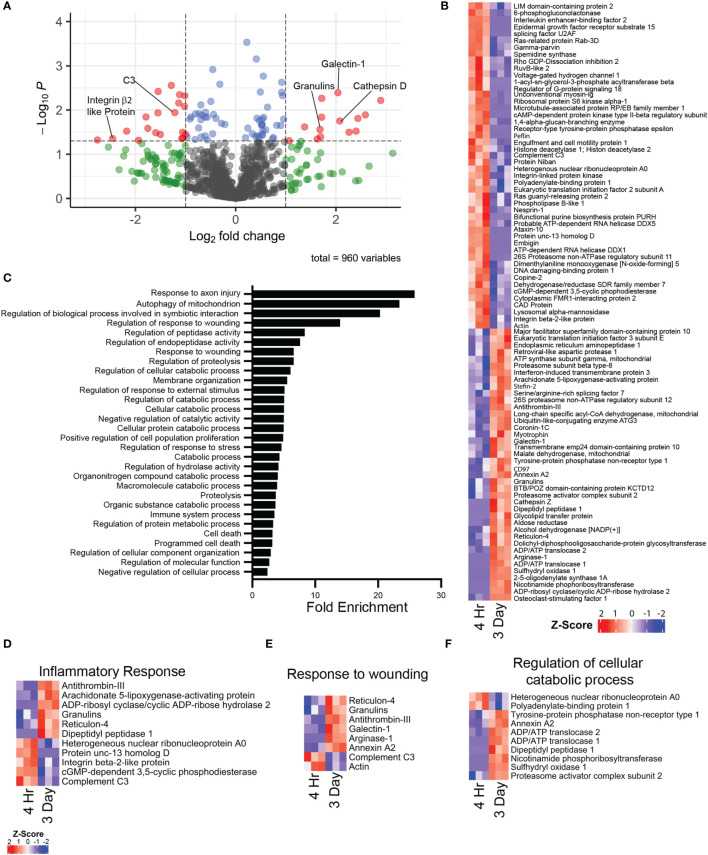
Mass spectrometry proteomic comparison of i.p. zymosan stimulated 4 hour and 3 day neutrophils (n = 3 mice per group). **(A)** Volcano plot illustrating differential protein expression between 4 hour and 3 day i.p. zymosan stimulation neutrophils. **(B)** Heat map of differentially expressed proteins between 4 hour and 3 day neutrophils as represented by row z-score. **(C)** Top differentially expressed GO biological process pathways based on proteins with increased expression in the 3 day neutrophils compared to 4 hour neutrophils. **(D–F)** Heat maps of selected GO biological process pathways, illustrating expression of proteins between 4 hour and 3 day neutrophils in the **(D)** inflammatory response pathway, **(E)** response to wounding pathway, and the **(F)** regulation of cellular catabolic process pathway.

Mass spectrometry proteomic analysis was performed on cell lysates of purified i.p zymosan stimulated 4hr NΦ and 3d NΦ. The 4h and 3d NΦ each had distinctive proteomic signatures (**Figure 6A, B**). 3d NΦ expressed high levels of arginase-1 protein, consistent with our previous data (16) and characterization of those cells as alternatively activated. Arginase-1 was undetectable in the 4hr NΦ samples (**Supplemental Table 1**). Pathway analysis indicates that 3d NΦ express relatively high levels of proteins associated with the response to axon injury and wound healing responses (**Figure 6C, E**). In particular, they expressed granulins and galectin-1, both of which have been associated with neuroprotection and axon regeneration (36, 37). Separately, 4h NΦ expressed higher levels of complement C3 and integrin β2-like protein which are associated with inflammation, while 3d NΦ expressed Cathepsin D which is associated with phagocytosis (**Figure 6A, D**). Additionally, 3d NΦ had high expression of cellular catabolic process GO pathway proteins associated with oxidative metabolism, supporting scRNA seq analysis indicating 3d NΦ are more metabolically active (**Figure 6F**).

## 4 Discussion

In this study, we performed an in-depth comparison of two populations of i.p zymosan modulated Ly6G^+^ neutrophils. On a population level, 3d NΦ collectively show features of alternative activation, an immature stage of development, and possesses neuroprotective/neuroregenerative properties; while 4h NΦ express a phenotype consistent with conventional, classically activated neutrophils and lacks neuroprotective/neuroregenerative properties. We also compared both zymosan modulated populations to naïve peripheral blood neutrophils. The results of the current study extend our previous observation that 3d NΦ are distinguished by expression of cell surface marker CD14 and cytosolic arginase expression with a more nuanced comparison of the neutrophil subsets. The transcriptomic analysis of the 3d NΦ populations identify transcripts associated with an immature stage of development (*Myc*, *Kit*, *Ly6a*, and *Ebf1)* (13, 27, 28), alternative activation (*Il4rα, Mrc*, and *Itga6*) (9), and a reparative phenotype (*Tgfb1*, *Hbegf*, *Spp1*, and *Igf)* (10, 16, 30, 38). Differential GO biological pathway analysis of the scRNAseq data confirmed that 3d NΦ upregulate genes involved in cell survival, immune modulation, and wound healing compared with either circulating blood neutrophils or 4h NΦ. Importantly, mass spectrometry proteomic analysis revealed candidate neuroprotective/regenerative factors produced by 3d NΦ that had not been previously identified (namely granulins and galectin-1) (36, 37).

Our characterization of a new neuroregenerative neutrophil subset adds to a growing body of literature that attests to the heterogeneity and functional sub-specialization of these cells. Neutrophils possess an array of signaling pathways that contribute to recruitment of additional immune cells, clearance of infection, and breakdown of injured tissue (5, 39, 40). Neutrophil recruitment and activation are also capable of inflicting tissue damage and prolonged inflammation. Neutrophil-driven inflammation is a common mechanism underlying many pathological conditions, including cardiovascular, acute respiratory, neurodegenerative, metabolic and autoimmune diseases, sepsis, and asthma (2, 6, 7, 11, 17, 18, 41).

In particular, zymosan-elicited neuroregenerative neutrophils are similar to other recently described subpopulations of neutrophils that are characterized by markers of immaturity through cell surface phenotype and nuclear morphology; as well as alternative activation based on expression of markers associated with M2-like macrophages such as arginase, mannose receptor, and IL-4rα expression. Alternatively activated neutrophils have been described to play immunoregulatory or reparative roles in murine models of cancer, chronic infection, and myocardial ischemia (5, 8–10, 16, 17). Additionally, alternatively activated neutrophils have been detected in ischemic brain tissue in rodent models of stroke, and their frequency correlates with increased neuronal survival, reduced infarct size, and enhanced clinical recovery (29). The extent to which the alternatively activated neutrophils characterized in these diverse models are biologically or developmentally related to one another, or share common mechanisms of action, remains to be determined. In both the zymosan stimulated i.o. neutrophils that are recruited after optic nerve crush, and the i.p. zymosan stimulated neutrophils, the commonly shared pathways include dectin-1 and toll like receptor 2 signaling based on previous work (16, 42). Additionally, there is a temporal relationship that is important to when these alternatively activated myeloid cells show up in response to zymosan. In both the i.o. and i.p zymosan stimulated neutrophils, early responding neutrophils have a classical activation phenotype, while a subset of the neutrophils that arrive later (3 days post zymosan injection) exhibit the alternative phenotype (16). The temporal relationship of myeloid cell chemotaxis is also important in the recruitment of reparative or immunosuppressive neutrophils after cardiovascular injury, stroke, certain infections, and cancer (2, 9, 10, 29, 43). Furthermore, the recruitment of alternatively activated neutrophils to sites of injury and the signaling pathways responsible for their alternatively activated state has yet to be elucidated. It is important to not only understand how these neutrophils function as reparative, but also to understand their chemotaxis to sites of injury, and the signaling involved resulting in their alternatively activated phenotype. Examining these questions could lead to novel immunotherapies to harness the myeloid immune response to improve recovery after neurological or other tissue injury.

## Data Availability Statement

The datasets presented in this study can be found in online repositories. The names of the repository and accession number for the RNA sequencing can be found below: NCBI Gene Expression Omnibus; accession number GSE20045. The repository for the proteomic data can be found below: MassIVE MSV000089342.

## Ethics Statement

The animal study was reviewed and approved by Ohio State University IACUC committee.

## Author Contributions

JS and AJ performed experiments. JA performed flow cytometry analysis. AJ. AS and AMV performed RNA-seq and proteomic analysis. AS and AJ wrote the manuscript and coedited it with the help of all the authors. BS and AS directed the studies. All authors contributed to the article and approved the submitted version.

## Funding

Financial support for this research was provided by the National Eye Institute (NEI), National Institutes of Health (R01EY029159 and R01EY028350 to BS; K08EY029362 to AS).

## Conflict of Interest

The authors declare that the research was conducted in the absence of any commercial or financial relationships that could be construed as a potential conflict of interest.

## Publisher’s Note

All claims expressed in this article are solely those of the authors and do not necessarily represent those of their affiliated organizations, or those of the publisher, the editors and the reviewers. Any product that may be evaluated in this article, or claim that may be made by its manufacturer, is not guaranteed or endorsed by the publisher.

## Supplementary Material

Supplementary Figure 1 Gating Strategy for flow cytometry analysis. **(A)** Representative flow cytometry gating strategy for i.p. zymosan stimulated neutrophils. **(B)** Representative flow cytometry demonstrating Ly6G purity of i.p. zymosan stimulated neutrophils after MACS bead sorting.

Supplementary Figure 2 Gene Ontology (GO) Pathway analysis of the most prevalent scRNA seq clusters. Top GO biological process pathways based on differentially expressed genes from each of clusters 0, 1, 2, 3, and 4.

Supplementary Table 1 Individual proteins and corresponding label free quantitation values for individual samples processed for mass spectrometry proteomic analysis. List of all proteins by protein ID and protein name, as well as corresponding average normalized LFQ values of each protein identified in the i.p. zymosan stimulated neutrophils submitted for mass spectrometry proteomics.

## References

[1] Giles DA Washnock-Schmid JM Duncker PC Dahlawi S Ponath G Pitt D. Myeloid Cell Plasticity in the Evolution of Central Nervous System Autoimmunity. Ann Neurol (2018) 83(1):131–41. doi: 10.1002/ana.25128 PMC5876132 29283442

[2] Tourki B Halade G. Leukocyte Diversity in Resolving and Nonresolving Mechanisms of Cardiac Remodeling. FASEB J (2017) 31(10):4226–39. doi: 10.1096/fj.201700109R PMC5602901 28642328

[3] Ginhoux F Jung S. Monocytes and Macrophages: Developmental Pathways and Tissue Homeostasis. Nat Rev Immunol (2014) 14(6):392–404. doi: 10.1038/nri3671 24854589

[4] Hirahara K Nakayama T. Cd4+ T-cell Subsets in Inflammatory Diseases: Beyond the Th1/Th2 Paradigm. Int Immunol (2016) 28(4):163–71. doi: 10.1093/intimm/dxw006 PMC4889886 26874355

[5] Nicolas-Avila JA Adrover JM Hidalgo A. Neutrophils in Homeostasis, Immunity, and Cancer. Immunity (2017) 46(1):15–28. doi: 10.1016/j.immuni.2016.12.012 28099862

[6] Gordon S. Phagocytosis: An Immunobiologic Process. Immunity (2016) 44(3):463–75. doi: 10.1016/j.immuni.2016.02.026 26982354

[7] Rumble JM Huber AK Krishnamoorthy G Srinivasan A Giles DA Zhang X. Neutrophil-Related Factors as Biomarkers in EAE and MS. J Exp Med (2015) 212(1):23–35. doi: 10.1084/jem.20141015 25559893 PMC4291533

[8] Veglia F Hashimoto A Dweep H Sanseviero E De Leo A Tcyganov E. Analysis of Classical Neutrophils and Polymorphonuclear Myeloid-Derived Suppressor Cells in Cancer Patients and Tumor-Bearing Mice. J Exp Med (2021) 218(4):1–20. doi: 10.1084/jem.20201803 PMC7879582 33566112

[9] Fridlender ZG Sun J Kim S Kapoor V Cheng G Ling L. Polarization of Tumor-Associated Neutrophil Phenotype by TGF-beta: “N1” Versus “N2” Tan. Cancer Cell (2009) 16(3):183–94. doi: 10.1016/j.ccr.2009.06.017 PMC2754404 19732719

[10] Horckmans M Ring L Duchene J Santovito D Schloss MJ Drechsler M. Neutrophils Orchestrate Post-Myocardial Infarction Healing by Polarizing Macrophages Towards a Reparative Phenotype. Eur Heart J (2017) 38(3):187–97. doi: 10.1093/eurheartj/ehw002 28158426

[11] Nakabo S Romo-Tena J Kaplan MJ. Neutrophils as Drivers of Immune Dysregulation in Autoimmune Diseases with Skin Manifestations. J Invest Dermatol (2022) 142(3 Pt B):823–33. doi: 10.1016/j.jid.2021.04.014 34253374

[12] Xie X Shi Q Wu P Zhang X Kambara H Su J. Single-Cell Transcriptome Profiling Reveals Neutrophil Heterogeneity in Homeostasis and Infection. Nat Immunol (2020) 21(9):1119–33. doi: 10.1038/s41590-020-0736-z PMC7442692 32719519

[13] Evrard M Kwok IWH Chong SZ Teng KWW Becht E Chen J. Developmental Analysis of Bone Marrow Neutrophils Reveals Populations Specialized in Expansion, Trafficking, and Effector Functions. Immunity (2018) 48(2):364–79 e8. doi: 10.1016/j.immuni.2018.02.002 29466759

[14] Giladi A Paul F Herzog Y Lubling Y Weiner A Yofe I. Single-Cell Characterization of Haematopoietic Progenitors and Their Trajectories in Homeostasis and Perturbed Haematopoiesis. Nat Cell Biol (2018) 20(7):836–46. doi: 10.1038/s41556-018-0121-4 29915358

[15] Paul F Arkin Y Giladi A Jaitin DA Kenigsberg E Keren-Shaul H. Transcriptional Heterogeneity and Lineage Commitment in Myeloid Progenitors. Cell (2016) 164(1-2):325. doi: 10.1016/j.cell.2015.12.046 28915372

[16] Sas AR Carbajal KS Jerome AD Menon R Yoon C Kalinski AL. A New Neutrophil Subset Promotes CNS Neuron Survival and Axon Regeneration. Nat Immunol (2020) 21(12):1496–505. doi: 10.1038/s41590-020-00813-0 PMC7677206 33106668

[17] Tsuda Y Takahashi H Kobayashi M Hanafusa T Herndon DN Suzuki F. Three Different Neutrophil Subsets Exhibited in Mice With Different Susceptibilities to Infection by Methicillin-Resistant Staphylococcus Aureus. Immunity (2004) 21(2):215–26. doi: 10.1016/j.immuni.2004.07.006 15308102

[18] Gadani SP Walsh JT Lukens JR Kipnis J. Dealing With Danger in the CNS: The Response of the Immune System to Injury. Neuron (2015) 87(1):47–62. doi: 10.1016/j.neuron.2015.05.019 26139369 PMC4491143

[19] Park J Shrestha R Qiu C Kondo A Huang S Werth M. Single-Cell Transcriptomics of the Mouse Kidney Reveals Potential Cellular Targets of Kidney Disease. Science (2018) 360(6390):758–63. doi: 10.1126/science.aar2131 PMC6188645 29622724

[20] Stuart T Butler A Hoffman P Hafemeister C Papalexi E Mauck WM 3rd. Comprehensive Integration of Single-Cell Data. Cell (2019) 177(7):1888–902 e21. doi: 10.1016/j.cell.2019.05.031 31178118 PMC6687398

[21] Han X Wang R Zhou Y Fei L Sun H Lai S. Mapping the Mouse Cell Atlas by Microwell-Seq. Cell (2018) 173(5):1307. doi: 10.1016/j.cell.2018.05.012 29775597

[22] Subramanian A Tamayo P Mootha VK Mukherjee S Ebert BL Gillette MA. Gene Set Enrichment Analysis: A Knowledge-Based Approach for Interpreting Genome-Wide Expression Profiles. Proc Natl Acad Sci U.S.A. (2005) 102(43):15545–50. doi: 10.1073/pnas.0506580102 PMC1239896 16199517

[23] Gu Z Eils R Schlesner M. Complex Heatmaps Reveal Patterns and Correlations in Multidimensional Genomic Data. Bioinformatics (2016) 32(18):2847–9. doi: 10.1093/bioinformatics/btw313 27207943

[24] Cao J Spielmann M Qiu X Huang X Ibrahim DM Hill AJ. The Single-Cell Transcriptional Landscape of Mammalian Organogenesis. Nature (2019) 566(7745):496–502. doi: 10.1038/s41586-019-0969-x 30787437 PMC6434952

[25] Hebert AS Prasad S Belford MW Bailey DJ McAlister GC Abbatiello SE. Comprehensive Single-Shot Proteomics With FAIMS on a Hybrid Orbitrap Mass Spectrometer. Anal Chem (2018) 90(15):9529–37. doi: 10.1021/acs.analchem.8b02233 PMC6145172 29969236

[26] Tyanova S Temu T Cox J. The MaxQuant Computational Platform for Mass Spectrometry-Based Shotgun Proteomics. Nat Protoc (2016) 11(12):2301–19. doi: 10.1038/nprot.2016.136 27809316

[27] Lee PY Wang JX Parisini E Dascher CC Nigrovic PA. Ly6 Family Proteins in Neutrophil Biology. J Leukoc Biol (2013) 94(4):585–94. doi: 10.1189/jlb.0113014 23543767

[28] Johansen LM Iwama A Lodie TA Sasaki K Felsher DW Golub TR. c-Myc is a Critical Target for C/Ebpalpha in Granulopoiesis. Mol Cell Biol (2001) 21(11):3789–806. doi: 10.1128/MCB.21.11.3789-3806.2001 PMC87031 11340171

[29] Cuartero MI Ballesteros I Moraga A Nombela F Vivancos J Hamilton JA. N2 Neutrophils, Novel Players in Brain Inflammation After Stroke: Modulation by the PPARgamma Agonist Rosiglitazone. Stroke (2013) 44(12):3498–508. doi: 10.1161/STROKEAHA.113.002470 24135932

[30] Todd L Volkov LI Zelinka C Squires N Fischer AJ. Heparin-Binding EGF-like Growth Factor (HB-EGF) Stimulates the Proliferation of Muller Glia-Derived Progenitor Cells in Avian and Murine Retinas. Mol Cell Neurosci (2015) 69:54–64. doi: 10.1016/j.mcn.2015.10.004 26500021 PMC4658256

[31] Duan X Qiao M Bei F Kim IJ He Z Sanes JR. Subtype-Specific Regeneration of Retinal Ganglion Cells Following Axotomy: Effects of Osteopontin and mTOR Signaling. Neuron (2015) 85(6):1244–56. doi: 10.1016/j.neuron.2015.02.017 PMC4391013 25754821

[32] Gambarotta G Ronchi G Geuna S Perroteau I. Neuregulin 1 Isoforms Could be an Effective Therapeutic Candidate to Promote Peripheral Nerve Regeneration. Neural Regener Res (2014) 9(12):1183–5. doi: 10.4103/1673-5374.135324 PMC4146285 25206780

[33] Eguchi J Kong X Tenta M Wang X Kang S Rosen ED. Interferon Regulatory Factor 4 Regulates Obesity-Induced Inflammation Through Regulation of Adipose Tissue Macrophage Polarization. Diabetes (2013) 62(10):3394–403. doi: 10.2337/db12-1327 PMC3781469 23835343

[34] Yang Q Li X Chen H Cao Y Xiao Q He Y. IRF7 Regulates the Development of Granulocytic Myeloid-Derived Suppressor Cells Through S100A9 Transrepression in Cancer. Oncogene (2017) 36(21):2969–80. doi: 10.1038/onc.2016.448 28092673

[35] Arora S Wang Y Jia Z Vardar-Sengul S Munawar A Doctor KS. Egr1 Regulates the Coordinated Expression of Numerous EGF Receptor Target Genes as Identified by Chip-on-Chip. Genome Biol (2008) 9(11):R166. doi: 10.1186/gb-2008-9-11-r166 19032775 PMC2614498

[36] Aalinkeel R Mahajan SD. Neuroprotective Role of Galectin-1 in Central Nervous System Pathophysiology. Neural Regener Res (2016) 11(6):896–7. doi: 10.4103/1673-5374.184455 PMC4962575 27482206

[37] Tao J Ji F Wang F Liu B Zhu Y. Neuroprotective Effects of Progranulin in Ischemic Mice. Brain Res (2012) 1436:130–6. doi: 10.1016/j.brainres.2011.11.063 22221732

[38] Kurimoto T Yin Y Habboub G Gilbert HY Li Y Nakao S. Neutrophils Express Oncomodulin and Promote Optic Nerve Regeneration. J Neurosci (2013) 33(37):14816–24. doi: 10.1523/JNEUROSCI.5511-12.2013 PMC3771038 24027282

[39] Kolaczkowska E Kubes P. Neutrophil Recruitment and Function in Health and Inflammation. Nat Rev Immunol (2013) 13(3):159–75. doi: 10.1038/nri3399 23435331

[40] Yang W Tao Y Wu Y Zhao X Ye W Zhao D. Neutrophils Promote the Development of Reparative Macrophages Mediated by ROS to Orchestrate Liver Repair. Nat Commun (2019) 10(1):1076. doi: 10.1038/s41467-019-09046-8 30842418 PMC6403250

[41] Mehrpouya-Bahrami P Moriarty AK De Melo P Keeter WC Alakhras NS Nelson AS. STAT4 is Expressed in Neutrophils and Promotes Antimicrobial Immunity. JCI Insight (2021) 6(14):1–8. doi: 10.1172/jci.insight.141326 PMC8410094 34138758

[42] Baldwin KT Carbajal KS Segal BM Giger RJ. Neuroinflammation Triggered by Beta-Glucan/Dectin-1 Signaling Enables CNS Axon Regeneration. Proc Natl Acad Sci U.S.A. (2015) 112(8):2581–6. doi: 10.1073/pnas.1423221112 PMC4345569 25675510

[43] Deerhake ME Reyes EY Xu-Vanpala S Shinohara ML. Single-Cell Transcriptional Heterogeneity of Neutrophils During Acute Pulmonary Cryptococcus Neoformans Infection. Front Immunol (2021) 12:670574. doi: 10.3389/fimmu.2021.670574 33995406 PMC8116703

