# Supplementary figures and images for: Characterization of Zymosan-Modulated Neutrophils With Neuroregenerative Properties
Source: Front Immunol. 2022 May 30;13:912193. doi: 10.3389/fimmu.2022.912193 (PMC9195616; doi:10.3389/fimmu.2022.912193)

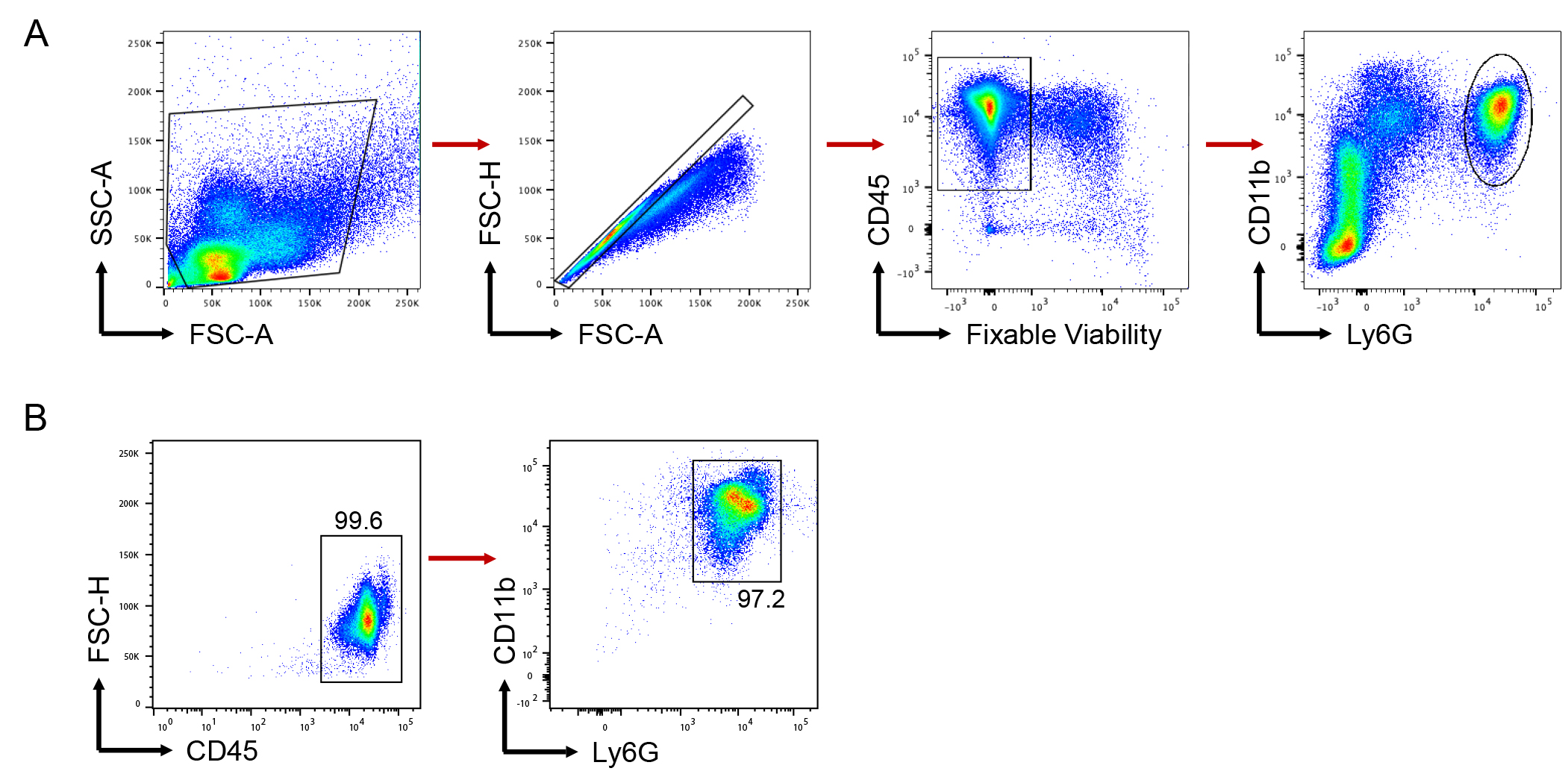

Supplement: Supplementary Figure 1 — Gating Strategy for flow cytometry analysis. (A) Representative flow cytometry gating strategy for i.p. zymosan stimulated neutrophils. (B) Representative flow cytometry demonstrating Ly6G purity of i.p. zymosan stimulated neutrophils after MACS bead sorting. [file Image_1.jpeg]

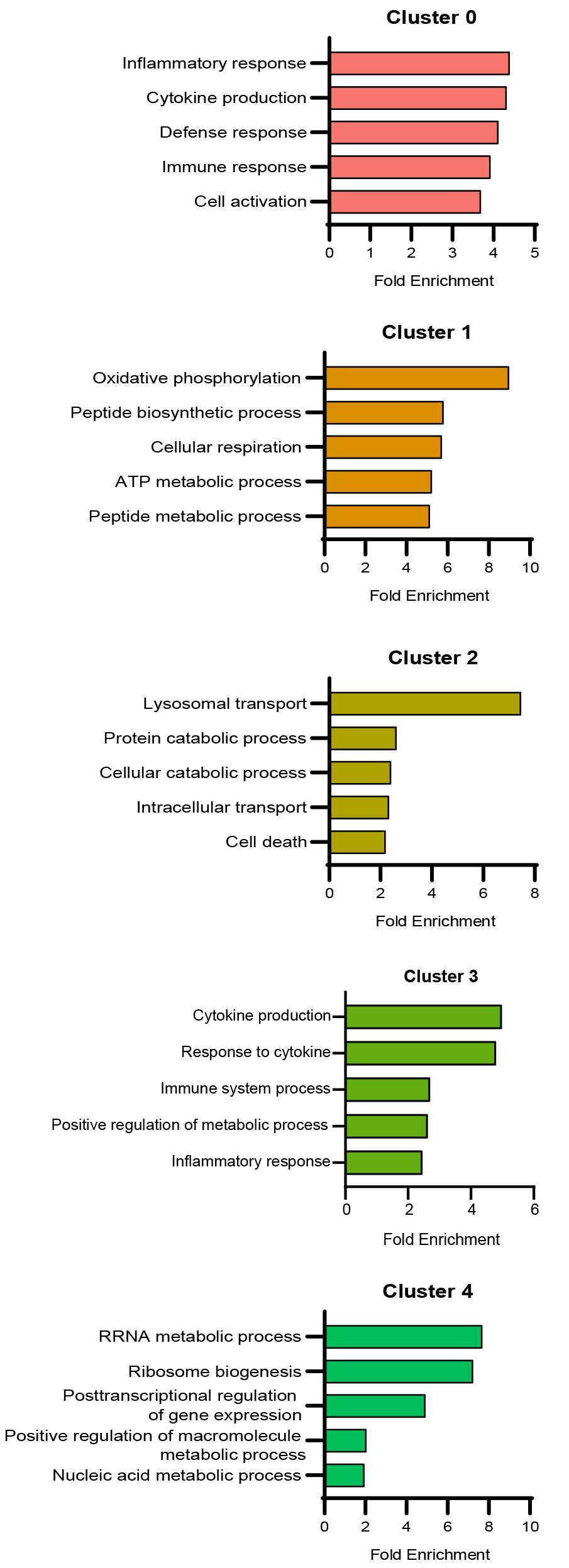

Supplement: Supplementary Figure 2 — Gene Ontology (GO) Pathway analysis of the most prevalent scRNA seq clusters. Top GO biological process pathways based on differentially expressed genes from each of clusters 0, 1, 2, 3, and 4. [file Image_2.jpeg]
